# Chemical Composition, Enantiomeric Distribution and Anticholinesterase and Antioxidant Activity of the Essential Oil of *Diplosthephium juniperinum*

**DOI:** 10.3390/plants11091188

**Published:** 2022-04-28

**Authors:** Melissa Salinas, James Calva, Luis Cartuche, Eduardo Valarezo, Chabaco Armijos

**Affiliations:** 1 Maestría en Química Aplicada, Universidad Técnica Particular de Loja, San Cayetano s/n, Loja 1101608, Ecuador; masalinas4@utpl.edu.ec; 2 Departamento de Química y Ciencias Exactas, Universidad Técnica Particular de Loja, Loja 1101608, Ecuador; jwcalva@utpl.edu.ec (J.C.); lecartuche@utpl.edu.ec (L.C.); bevalarezo@utpl.edu.ec (E.V.)

**Keywords:** *D. juniperinum*, essential oil, GC/MS, GC/FID, enantiomers, AChE, BuChE, antioxidant activity

## Abstract

The aim of this study was to extract and identify the chemical compounds of *Diplosthephium juniperinum* essential oil (EO) from Ecuador and to assess its anticholinesterase and antioxidant properties. The EO chemical composition was determined by GC–MS. A total of 74 constituents of EO were identified, representing 97.27% in DB-5ms and 96.06% in HP-INNOWax of the total EO. The major constituents (>4.50%) identified were: α-pinene (21.52, 22.04%), geranyl acetate (10.54, 7.78%), silphiper-fol-5-ene (8.67, 7.38%), α-copaene (8.26, 8.18%), 7-*epi*-silphiperfol-5-ene (4.93, 5.95%), and germacrene D (4.91, 6.00%). Enantioselective analysis of the volatile fraction of *D. juniperinum* showed: (+)-α-pinene as a pure enantiomer and 5 pairs of enantiomeric compounds. Among them, (−)-β-Pinene and (−)-Germacrene D presented a high enantiomeric excess of 93.23 and 84.62%, respectively, while (−)-α-Thujene, (−)-Sabinene and (S)-4-Terpineol with a lower enantiomeric excess of 56.34, 47.84 and 43.11%, respectively. A moderate inhibitory effect was observed for Acetylcholinesterase (AChE) and Butyrylcholinesterase (BuChE) enzymes with IC_50_ values of 67.20 ± 7.10 and 89.00 ± 9.90 µg/mL, respectively. A lower antioxidant potential was observed for the EO measured through DPPH and ABTS radical scavenging assays with SC_50_ values of 127.03 and >1000 µg/mL, respectively. To the best of our knowledge, this is the first report of the chemical composition, enantiomeric distribution and, anticholinesterase and antioxidant potential of the EO of *D. juniperinum*. As future perspective, further in-vivo studies could be conducted to confirm the anticholinesterase potential of the EO.

## 1. Introduction

Asteraceae family is the largest group of vascular plants in the world and is composed mainly of flowering plants (angiosperms). Also called Compositae, the Asteraceae family comprises approximately 32,205 species belonging to 1911 plant genera [1], and grouped into 13 subfamilies [2,3]. Many species of this family are mainly herbaceous plants, however it can include trees, shrubs and sub-shrubs to vines [4]. Asteraceae occurs on all continents except Antarctica. On a global scale, the diversity of Asteraceae reported, is distributed as follows: South America (6316 species), Asia (6016 species), North America (5404 species), Africa (4631 species), Europe (2283 species), Oceania (1444 species), and the Pacific Islands (174 species) [5].

Despite its large number of species, a small number of them have been used for human and animal consumption as weeds (*Bidens*, *Cirsium*, *Hypochaeris* and *Sonchus* genera) [6,7], for its toxic and insecticidal properties, in gardening, for ornamental use (*Aster*, *Bellis*, *Cosmos*, *Chrysanthemum, Gazania* and *Gerbera* genera), in the food industry as oil plants (*Helianthus annus* and *Carthamus tinctorius*), in the pharmaceutical (secondary metabolites with important biological activities) [8]. Important medicinal plants such as *Matricaria chamomilla*, *Artemisia absinthium* and *Tussilago farfara* belongs to this family [9]. Numerous members of the Asteraceae family are important as aromatic plants, from which essential oil (EO) can be extracted. These EOs are used in alternative and traditional medicine, and as ingredients for pharmaceutical and cosmetics industries. The Asteraceae EOs have a broad spectrum of bioactivity biological owing to the presence of active chemical compounds [10]. However, in Asteraceae family, as well as, in Boraginaceae and Fabaceae families, have been reported a series of chemical compound of alkaloid nature that are toxic for livestock and humans. These natural compounds named pyrrolizidine alkaloids are natural toxins occurring in Asteraceae family, extracted mainly with organic solvents from the plant material [11], in contrast to EOs that are a mixture of volatile compounds of terpene nature, that have a low or minimum toxicity with some exceptions, as safrole which is a natural compound present in EOs from *Piper* genus [12] or extracted commonly from *Sassafras* genus [13].

Of the 18,500 species of vascular plants registered in Ecuador, orchids are the most diverse, with 4200 species, followed by Asteraceae with 918 species, which belong to 217 genera, 7 of them endemic [14]. In addition, in Ecuador this family is recognized for the number of endemic species, with 370 specimens, located in second after orchids. Endemic Asteraceae are mainly shrubs (195 species) and herbs (97 species). The Ecuadorian Andes are the center of diversity and endemism in this family, although there are species in the Amazon, Coast and Galapagos (the four natural regions of Ecuador). Of the endemic species found in Ecuador, 32 are exclusive to Galapagos [15]. Asteraceae species exhibit a wide altitude spectrum from near sea level to 5000 m of altitude. In Ecuador the diversity of this family increases from 2000 to 3000 m a.s.l, registering a maximum between 2900 to 3000 m a.s.l. [9].

*Diplostephium* is a genus of trees, shrubs, and subshrubs that are part of the flora of the upper limit of the Andean forests, paramos, jalcas and punas in the neotropical mountains. Currently this genus is composed of 111 accepted species names [16], distributed from Costa Rica to Chile in high elevation cloud forests (2500–3000 m), puna habitats (3800–4200 m) and paramos (3000–4500 m) [17]. In Latin America, 63 species have been reported for Colombia, 39 for Peru, 26 for Ecuador, 10 for Venezuela, three for Chile and one for Bolivia [18]. *Diplostephium juniperinum* Cuatrec (Kunth), known as “monte de baño” (bath grass) is an endemic shrub of Ecuador, distributed in the Andean regions between 2000 to 3400 m a.s.l., especially in the Andean provinces of Azuay and Loja [14] and is used by indigenous Saraguro (Loja, province) in postpartum herbal bath [19]. This species has been found only in Ecuador, its natural habitat is subtropical or tropical moist montane forests. The *D. juniperinum* plant is a 0.8 m tall shrub which topped is round. This species has branches densely compacted, bracts green with reddish purple tinge, disk flowers dull yellow, ray florets white and tipped with pale lavender below [20].

Ecuador is considered a megadiverse country because has many species per unit surface area. Currently, this country occupies the sixth position worldwide as a biodiversity hotspot [21]. However, the fact that there are few studies of its aromatic plant species, especially of the aromatic species of the Asteraceae family, and that study of the *D. juniperinum* EO having not been previously reported in the literature have stimulated our interest in investigating the EO extracted from this species. For that reason, the aim of this research was to determine the chemical composition, and enantiomeric distribution of the EO of *D. juniperinum*, as well as, to assess its antioxidant and anticholinesterase properties and thus, contribute to the phytochemical characterization of *Diplostephium* species in Ecuador. In addition, the search for new natural products or compounds with biological interest is of relevance for the pharmaceutical and cosmetic industry nowadays.

## 2. Results

### 2.1. Physical Properties

Through hydrodistillation from fresh aerial parts of *D. juniperinum*, a pale-yellow EO was obtained, with a low extraction yield of 0.12 ± 0.01% (*w*/*w*), a relative density of 0.79 ± 0.02 gr/mL, refractive index [n^20^] 1.48 ± 0.01 and a specific rotation [α]_D_^20^ = −34.18 ± 0.01°.

### 2.2. Chemical Composition

**Figure 1:**
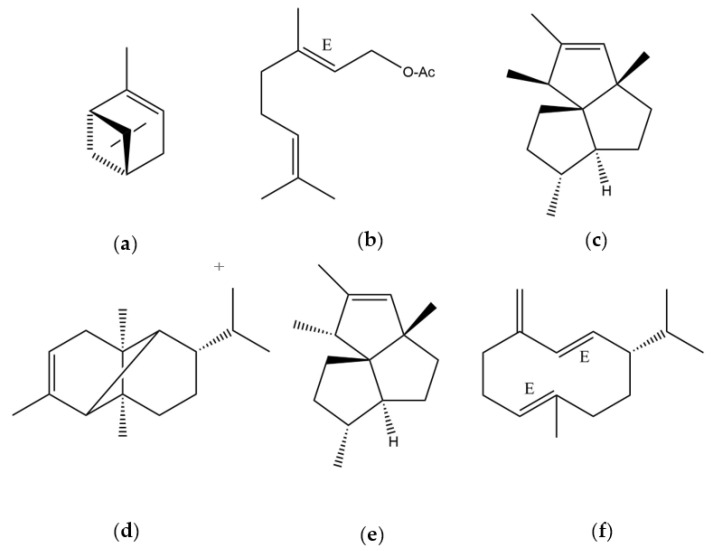
Principal compounds of essential oil of *D. juniperinum:* (**a**) α-pinene; (**b**) geranyl acetate; (**c**) silphiperfol-5-ene; (**d**) α-copaene; (**e**) 7-*epi*-silphiperfol-5-ene; (**f**) germacrene D.

A total of 74 constituents were identified, representing 97.27% in DB-5ms and 96.06% in HP-INNOWax of the total EO composition. The main constituents (>4.50%) identified were: α-pinene (21.52, 22.04%) (a), geranyl acetate (10.54, 7.78%) (b), silphiperfol-5-ene (8.67, 7.38%) (c), α-copaene (8.26, 8.18%) (d), 7-epi-silphiperfol-5-ene (4.93, 5.95%) (e), and germacrene D (4.91, 6.00%) (f) (Figure 1 and Table 1).

Sesquiterpene (SH) and monoterpene hydrocarbons (MH) predominated in the chemical composition of the *D. juniperinum* EO. The percentages of SH were 45.34% and 44.80% in DB-5ms andHP-INNOWax, respectively. MH in DB-5ms column represented 34.53% and in HP-INNOWax 35.61%. Oxygenated monoterpenes represented 13.21% (DB-5ms) and 9.92% (HP-INNOWax), followed by oxygenated sesquiterpenes with 3.24% (DB-5ms) and 4.17% (HP-INNOWax) and finally, other compounds with 0.95% in DB-5ms and 1.39% in HP-INNOWax.

### 2.3. Enantiomeric Composition

Enantioselective analysis of the volatile fraction of *D. juniperinum* showed: (+)-α-pinene as pure enantiomer and 5 pairs of enantiomeric compounds, among them; (−)-β-Pinene and (−)-Germacrene D reported a high enantiomeric excess of 93.23 and 84.62%, respectively, while (−)-α-Thujene, (−)-Sabinene and (S)-4-Terpineol with a lower enantiomeric excess of 56.34, 47.84 and 43.11%, respectively (Table 2).

### 2.4. Anticholinesterase Activity

In this study, we evaluated for the first time the anti-cholinesterase activity of *D. juniperinum* EO by measuring the rate of reaction. Results showed a moderate inhibition effect with IC_50_ values of 67.20 ± 7.10 and 89.00 ± 9.90 µg/mL against AChE and BuChE, respectively. Donepezil hydrochloride was used as a positive control and their value of IC_50_ is presented in Table 3.

### 2.5. Antioxidant Activity

The results obtained for DPPH and ABTS radical scavenging of the *D. juniperinum* EO as presented in Table 4, and expressed as the concentration of the EO that scavenge or decrease the concentration of the radical at 50% (SC_50_). Trolox was used as a positive control.

## 3. Discussion

Average yields of EO were calculated based on the fresh plant material of the aerial parts of *D. juniperinum* was similar with *D. antioquense* EO with 0.16% and much higher that the reported in *D. rosmarinifolius* with a very low yield 0.0045% [33].

*D. juniperinum* species does not present previous chemical studies of the volatile fraction, however, the EOs obtained from plants of the same genus such as *D. antioquense* and *D. rosmarinifolius* collected in Colombia were analyzed by GC/MS and GC/FID determining to β-copaene (17.78%), (*Z*)-para-mentha-2,8-dien-1-ol (14.29%), β-pinene (13.75%), δ-cadinene (11.42%) and *E*-caryophyllene (6.54%) as majority constituents of *D. antioquense*, while in *D. rosmarinifolius* EO were found to *E*-caryophyllene (16.07%), 1*R*-α-pinene (13.79%), δ-cadinene (8.54%), limonene (8.23%), α-caryophyllene (8.15%) and γ-terpineol (8.11%) [33].

Two monoterpenes identified as main constituents of EO of *D. juniperinum*, α-pinene and geranyl acetate were isolated and reported with biological activities in other studies; α-pinene exhibits antinociceptive [34], anti-inflammatory [35,36,37], antidepressant [38] and antioxidant properties [39], while, geranyl acetate has shown significant anti-Candida potential [40] and antinociceptive properties [41].The rare sesquiterpenes silphiperfol-5-ene and 7-*epi*-silphiperfol-5-ene were found in *Pteronia* genus of the Asteraceae family [27], but there are no reports of their isolation or biological activity.

Germacrene D, is one of the main components identified in the *D. juniperinum* EO, and the enantiomer (−)-germacrene D was found with an *e.e.* of 92.31 %. Biologically, this sesquiterpene exerted promising results, potentially influence in the attraction and oviposition of females of the species *Heliothis virescens* [42]. Chiral compounds have great importance for the identification of adulterations due to EOs have different proportions of each enantiomer [43] and this enantiomeric characterization is also important in the olfactory profile [44].

Natural acetylcholinesterase inhibitors, such as galantamine, are usually used in the pharmacological industry as a drug to treat Alzheimer’s disease [45], the search for future AChE and BuChE inhibitors guarantee the alleviation of symptoms related to the aforementioned disease and the reduction of mortality rates [46]. Several studies on the anticholinesterase activity of EOs and almost none on their main components showed that EOs are complex mixtures and their final activities are due to the combined effects of the all components [47], therefore, the inhibitory activity of the EO is probably the result of a complex interaction of its chemical components, producing synergistic or antagonistic inhibitory responses [48].

Anti-cholinesterase effect of EO from *Diplostephium* genus, has not been reported, the monoterpenes are the kind of compounds predominant in them. As mentioned by Aazza and collaborators [49] the α-pinene, limonene and sabinene, are responsible for the anticholinesterase effect. Additionally, (+)-α-Pinene as reported by Miyazawa and Yamafuji [50], presented an IC_50_ of 0.40 mM against acetylcholinesterase and, this compound was identified in the EO of *D. juniperinum* at a concentration of 21% and enantiomerically pure, which could explain the moderate effect observed for this EO against AChE and BuChE enzymes. Therefore, it is important to know the main constituents of the EOs, their proportion and chiral composition because they are the ones that give their biological potential.

A literature review on the *Diplostephium* genus indicates that few studies have been conducted on its species, one of them is on the ethanolic extract of *D. phylicoides*, which shows a high antioxidant activity (IC_50_ = 13.80 µg/mL) attributed to the presence of flavonoids in its composition [51].

In other study, α-pinene reported a lower antioxidant effect, with an IC_50_ of 12.57 ± 0.18 mg/mL [52]. Similar results for the EO of *D. juniperinum* for ABTS and DPPH assays with an SC_50_ of ca. 120 µg/mL and >1000 µg/mL were observed. The importance of knowing the antioxidant properties of EO is due to their implication in counteracting the harmful effects on biological entities by free radicals or reactive oxygen species [53].

Several studies have demonstrated that extracts of Astareaceae species have a positive impact on human health, thanks to their anti-inflammatory, antimicrobial and antioxidant, and antimicrobial [54]. Recently, species of the Asteraeceae family have been considered as a sustainable planning tool in cities for their phytoremediation properties as air pollutant removal, soil protection, shaping landscapes, etc. [55]. Further studies can be conducted to validate the anticholinesterase effect in in vivo studies, however, the low yield obtained for this species could difficult such approximation. In order to obtain a better amount needed for in vivo assays, oil extraction optimization studies could be carried out, including the study of intrinsic and extrinsic parameters related to the species, such as plant age, phenological stage, soil type, amount of shade and season of the year when the species is harvested [56]. This further research could complement the current one.

## 4. Materials and Methods

### 4.1. Materials

Methanol and dichloromethane from analytical HPLC grade, anhydrous sodium sulphate, 2,2′-azinobis-3-ethylbenzothiazoline-6-sulfonic acid (ABTS), 2,2-diphenyl-1-picrylhydrazyl (DPPH), Butyrylcholinesterase from equinum serum, Acetylcholinesterase from *Electrophorus electricus*, phosphate buffered saline, Ellman’s reagent (5,5′-dithiobis(2-nitrobenzoic acid), Acetylthiocoline iodide, donepezil hydrochloride, were purchased from Sigma-Aldrich (San Luis, MO, USA).The standard aliphatic hydrocarbons were purchased from ChemService (West Chester, PA, USA). Helium was purchased from INDURA (Guayaquil, Ecuador). All chemicals were of analytical grade and used without further purifications.

### 4.2. Plant Material

Leaves, stems and flowers of *D. juniperinum* were collected in October 2020 in Las Antenas sector, at the border between Saraguro and San Lucas, Loja province, at an altitude of 3210 m a.s.l. and located at 9,593,252 N, 696,030 E coordinates. The plant material collected under permit MAE-DBN-2016-048 granted by the Ministry of Environment of Ecuador (MAE), was identified and classified by José Miguel Andrade, botanist at UTPL. A specimen sample was deposited at the Herbarium of the Universidad Técnica Particular de Loja (HUTPL) with voucher code PPN-as-057.

### 4.3. Distillation of the Essential Oil

The EO from fresh aerial parts of *D. juniperinum* was extracted by steam hydrodistillation in a Clevenger-type apparatus for approximately 3 h. Three distillations were carried out with 1300, 1320 and 1410 g of fresh plant material, respectively. After obtaining the EO it was separated from the aqueous phase and dried with anhydrous sodium sulfate, filtered and stored in an amber sealed vial at −4 °C, until its analytical and biological assays. The procedure was performed three times [57].

### 4.4. Physical Properties of Essential Oil

The relative density, refractive index and optical rotation of the EO of *D. juniperinum* were determined in triplicate at 20 °C. The relative density was determined according to the AFNOR NF T 75-11 method (equivalent to ISO 279: 1998, using a pycnometer of 1 mL capacity and an analytical balance (Mettler AC 100), the refractive index according to AFNOR method NF 75-112 (ISO 280:1998) in a refractometer model ABBE (BOECO, Hamburg, Germany). The specific optical rotation was determined with the ISO 592-1998 standard method in an automatic polarimeter (Hanon P-810) [23].

### 4.5. Chemical Characterization of Essential Oil

#### 4.5.1. Sample Preparation of EO

Quantitative and qualitative characterization of EO from *D. juniperinum* required sample preparation of the volatile fractions. Ten µL of EO was diluted in 990 µL in dichloromethane (CH_2_Cl_2_) obtaining a 1:100 *v*/*v* solution. The samples were used in the chemical analyses described below [25].

#### 4.5.2. Qualitative and Quantitative Analysis

Qualitative identification was performed using the analytical technique of Gas Chromatography coupled to Mass Spectrometry (GC/MS). One µL of each sample was injected in duplicate in split mode (40:1) at 20 °C into an Agilent Technologies model 6890N gas chromatograph (GC) with an autoinjector model 7683 and a mass spectrometer model 5973 INERT (Santa Clara, CA, USA). The GC equipment operates in electron-ionization mode at 70 eV, with helium as carrier gas (1.00 mL/min in constant flow), the GC oven operated with temperature ramp from 60 °C to 250 °C with a gradient of 3 °C/min and the ion source at 250 °C. Additionally, the capillary columns DB-5ms (5%-phenyl-methyl polysiloxane, 30  m × 0.25  mm i.d., 0.25  μm film thickness;) and HP-INNOWax, (polyethylene glycol, 30 m × 0.25  mm i.d., 0.25  μm film thickness both purchased from J & W Scientific, Folsom, CA, USA, were used. The procedure was performed for triplicate.

The identification of the aromatic compounds was performed by comparison of the mass spectra and the linear retention index (LRI) with those reported in literature. The LRI was determined experimentally according to Van Den Dool and Krats [58], for which it was necessary to inject a homologous series of C_9_ to C_24_ alkanes in the same conditions of the EO.

Quantitative analysis of the EO of *D. juniperinum* was performed using a gas chromatography coupled to a flame ionization detector (GC/FID). The previously prepared samples were injected under the same analytical conditions as the qualitative GC/MS method, and the chromatography columns were the same. The percentage of aromatic compounds was determined by comparing the GC peaks with the total area of the identified peaks [59]. A calibration curve was built for each column as previously described by Gilardoni et al. [60], using isopropyl caproate (0.6, 1.8, 4.3, 8.3, 16.8, and 34.3 mg of isopropyl caproate in 10 mL of cyclohexane) and n-nonane (7 mg) as calibration standard and internal standard respectively. The LOD (0.4 μg/mL) and LOQ (1.2 μg/mL) were stablished. Both calibration curves generated a correlation coefficient of 0.995.

#### 4.5.3. Enantioselective Analysis of Essential Oil

Enantiomeric compounds present in the EO of *D. juniperinum* were determined by GC/MS on a capillary column with 2,3-diethyl-6-tert-butyldimethylsilyl-β-cyclodextrin stationary phase. The injection conditions used were the same in GC/MS. In addition, enantiomerically pure standards were injected under the same conditions to determine the elution order of the EO enantiomers [61].

### 4.6. AChE and BuChE Inhibition Spectrophotometric Analysis

Cholinesterase (ChEs) inhibition of EO was determined for the enzymes (i) acetylcholinesterase (AChE) and (ii) butyrylcholinesterase (BuChE). The procedure was followed as described by Ellman et al. [62] and Calva et al. [57]. Phosphate buffered saline (pH = 7.4), DTNB (5,5′-dithiobis-(2-nitrobenzoic acid) ion (1.5 mM) a reagent that reacts with thiocholine to give the yellow coloration and the EO sample in DMSO (1% *v*/*v*) were prepared. The reaction of DTNB is monitored by measuring its absorption at 412 nm. AChE, from Electrophorus electricus (Sigma-Aldrich, C3389, St. Louis, MO, USA) and BuChE, from horse serum, (Sigma-Aldrich, SRE020, St. Louis, MO, USA) are dissolved in PBS (pH = 7.4) at 24 mU/mL. Preincubation was carried out for 10 min and acetylcholine iodide (1.5 mM) is added to initiate the reaction. The reaction is monitored for 30 min at 30 °C in a PherastarFS detection system (BMG Labtech). Inhibitory concentration (IC_50_) values were calculated in the online package GNUPLOT (www.ic50.tk, www.gnuplot.info) (accessed on 1 March 2022). Measurements were performed by triplicate. The reference drug inhibitor of ChEs was Donepezil, for AChE and BuChE with an IC_50_ value of 100 nM and 8500 nM, respectively. False positives are not excluded for high concentrations (>100 ug/mL) of amine or aldehyde compounds [59].

### 4.7. Antioxidant Spectrophotometric Analysis

#### 4.7.1. DPPH Assay

The DPPH radical scavenging assay was developed according to the metodologhy proposed by Thaipong et al. [63] with slight modifications, using 2,2-diphenyl-1-picrylhydryl free radical (DPPH-). A working solution was prepared dissolving 24 mg of DPPH in 100 mL methanol and was stabilized in an EPOCH 2 microplate reader (BIOTEK, Winooski, VT, USA) at 515 nm until an absorbance of 1.1 ± 0.01 was reached. The antiradical reaction between EO and free radical was performed at different concentrations of EO (1, 0.5 and 0.25 mg/mL). In a 96-microwell plate, 270 µL of DPPH adjusted working solution and 30 µL of EO sample was placed. The reaction was monitored at 515 nm for 60 min at room temperature. Trolox and methanol were used as positive control and blank control, respectively. The results were expressed as SC_50_ (scavenging concentration of the radical at 50%) and calculated according to the corresponding curve fitting of data with GraphPadPrism v.8.0.1. Measurements were performed in triplicate.

#### 4.7.2. ABTS Assay

The antioxidant power measured against ABTS^•+^ cation (2,2′-azinobis-3-ethylbenzothiazoline-6-sulfonic acid) was determined as reported by Arnao et al. [64] and Thaipong et al. [63] with slight modifications as described. Briefly, the assay started with the preparation of a stock solution of the radical by reacting equal volumes of ABTS (7.4 µM) and potassium persulfate (2.6 µM) for 12 h under stirring. The standard solution was prepared by dissolution in methanol to an absorbance of 1.1 ± 0.02 measured at 734 nm in an EPOCH 2 microplate reader (BIOTEK, Winooski, VT, USA). The antiradical reaction was evaluated over a time of 1 h in the dark at room temperature by plating 270 µL of ABTS working adjusted solution and 30 µL of EO from *D*. *juniperinum* at different concentrations (1, 0.5 and 0.25 mg/mL). Trolox and methanol were used as positive control and blank control, respectively. The results were expressed as SC_50_ (scavenging concentration of the radical at 50%) and calculated according to the corresponding curve fitting of data with GraphPadPrism v.8.0.1. Measurements were performed in triplicate

## 5. Conclusions

The fresh aerial parts of *D. juniperinum* afforded, an essential oil in quite a low yield (0.12% by weight). The EO obtained was composed exclusively of sesquiterpenes and monoterpenes hydrocarbons, whose major constituents were α-pinene (about 22%) and geranyl acetate (about 10%). The enantioselective analysis showed (+)-α-pinene as a pure enantiomer and 5 pairs of enantiomeric compounds. The EO also manifested a moderate inhibition activity against AChE and BuChE and a lower antioxidant potential was observed for the EO measured through DPPH and ABTS radical scavenging assays. As future perspective, further in-vivo studies could be conducted to confirm the anticholinesterase potential of the EO. In addition, this genus that reported bioactive compounds, could be of interest for the development of new applications such as in the food industry, as enrichment of the food matrix to enhance their beneficial properties and also the substitution of synthetic antioxidants.

## Figures and Tables

**Table 1:** Chemical composition of *D. juniperinum* essential oil.

N°	Compound	DB-5ms					HP-INNOWax				
		LRI ^a^	LRI ^b^	Ref.	%	SD	LRI ^a^	LRI ^b^	Ref.	%	SD
1	α-Thujene	919	924	[22]	0.17	0.04	-	-	-	-	-
2	α-Pinene	926	932	[22]	21.52	3.76	1062	1066	[23]	22.04	4.06
3	Camphene	939	946	[22]	0.54	0.05	1083	1084	[23]	0.59	0.06
4	Thuja-2,4(10)-diene	943	953	[22]	0.20	0.05	1124	1122	[24]	0.29	0.03
5	Sabinene	964	969	[22]	1.74	0.22	1120	1120	[25]	1.83	0.13
6	β-Pinene	969	974	[22]	3.54	0.42	1109	1108	[25]	3.68	0.52
7	Myrcene	986	988	[22]	0.68	0.14	-	-	-	-	-
8	α-Phellandrene	1004	1002	[22]	0.75	0.07	1161	1163	[25]	1.38	0.16
9	α-Terpinene	1013	1014	[22]	0.34	0.16	1176	1185	[26]	0.28	0.01
10	ρ-Cymene	1020	1020	[22]	0.39	0.48	1273	1270	[25]	1.39	0.17
11	Limonene	1024	1024	[22]	1.97	0.54	1198	1199	[25]	2.07	0.13
12	1,8-Cineole	1028	1026	[22]	0.77	0.18	1208	1206	[25]	0.95	0.01
13	*Z*-β-Ocimene	-	-	-	-	-	1236	1236	[25]	0.11	0.02
14	*E*-β-Ocimene	1043	1044	[22]	2.46	0.26	1254	1253	[23]	2.72	0.33
15	ϒ-Terpinene	1052	1054	[22]	0.39	0.11	1244	1244	[25]	0.46	0.01
16	Terpinolene	1079	1086	[22]	0.23	0.14	1284	1290	[27]	0.17	0.01
17	n-Nonanal	1106	1100	[22]	0.22	0.04	-	-	-	-	-
18	α-Campholenal	1123	1122	[22]	0.34	0.09	-	-	-	-	-
19	*trans*-Pinocarveol	1135	1135	[22]	0.31	0.10	-	-	-	-	-
20	Borneol	1167	1165	[22]	0.31	0.16	-	-	-	-	-
21	Terpinen-4-ol	1176	1174	[22]	0.75	0.16	1609	1600	[27]	0.66	0.01
22	Myrtenal	1189	1195	[22]	0.24	0.07	1623	1631	[24]	0.13	0.01
23	Thymol, methyl ether	1227	1232	[22]	0.28	0.08	-	-	-	-	-
24	Silphiperfol-5-ene	1331	1326	[22]	8.67	0.40	1464	1495	[27]	7.38	2.45
25	α-Cubebene	1338	1348	[22]	0.15	0.08	1449	1460	[26]	0.23	0.01
26	7-*epi*-Silphiperfol-5-ene	1345	1345	[22]	4.93	0.30	1437	1424	[27]	5.95	0.99
27	Cyclosativene	1354	1369	[22]	0.20	0.00	-	-	-	-	-
28	α-Copaene	1365	1374	[22]	8.26	0.55	1479	1483	[23]	8.18	1.40
29	β-Cubebene	1377	1387	[22]	0.99	0.01	1528	1531	[23]	0.34	0.01
30	Geranyl acetate	1383	1379	[22]	10.54	0.55	1766	1761	[25]	7.78	1.07
31	α-Gurjunene	1392	1409	[22]	1.90	0.16	1514	1520	[23]	2.24	0.12
32	Pinocarvone	-	-	-	-	-	1563	1559	[28]	0.17	0.04
33	β-Gurjunene	-	-	-	-	-	1574	1559	[28]	0.58	0.67
34	*E*-Caryophyllene	1414	1419	[22]	2.09	0.05	1580	1586	[23]	1.07	1.33
35	β-Copaene	1425	1430	[22]	0.16	0.06	-	-	-	-	-
36	Aromadendrene	-	-	-	-	-	1615	1613	[25]	0.17	0.06
37	*cis*-Muurola-3,5-diene	1444	1448	[22]	0.27	0.14	-	-	-	-	-
38	α-Humulene	1449	1452	[22]	1.22	0.14	1652	1657	[23]	1.09	0.11
39	allo-Aromadendrene	1443	1458	[22]	0.19	0.06	1626	1633	[23]	0.37	0.25
40	*cis*-Cadina-1(6),4-diene	1469	1461	[22]	0.43	0.10	-	-	-	-	-
41	*cis*-Muurola-4(14),5-diene	1474	1465	[22]	1.40	0.16	1646	1648	[29]	0.29	0.02
42	*cis*-Verbenol	-	-	-	-	-	1661	1663	[30]	0.22	0.09
43	Germacrene D	1477	1484	[22]	4.91	0.90	1691	1697	[25]	6.00	0.92
44	β-Selinene	1482	1489	[22]	0.96	0.06	1698	1702	[29]	1.04	0.02
45	ϒ-Muurolene	1485	1478	[22]	0.51	0.25	1687	1681	[26]	0.11	0.02
46	Viridiflorene	-	-	-	-	-	1679	1686	[26]	0.27	0.03
47	α-Selinene	-	-	-	-	-	1704	-	-	0.65	0.18
48	α-Muurolene	-	-	-	-	-	1711	1717	[23]	1.69	0.11
49	Bicyclogermacrene	1490	1500	[22]	1.11	0.67	1716	1723	[25]	1.05	0.13
50	α-Amorphene	1497	1483	[22]	1.49	0.40	1674	1679	[31]	1.40	0.15
51	Germacrene A	1500	1508	[22]	0.37	0.21	-	-	-	-	-
52	Silphiperfolan-6-α-ol	1503	1507	[22]	0.17	0.04	-	-	-	-	-
53	*trans*-Muurola-4(14),5-diene	1498	1493	[22]	0.68	0.01	-	-	-	-	-
54	δ-Cadinene	1517	1522	[22]	3.96	1.05	1745	1750	[29]	4.00	1.04
55	*trans*-Cadina1,4-diene	1528	1533	[22]	0.15	0.04	-	-	-	-	-
56	α-Cadinene	1532	1537	[22]	0.13	0.03	-	-	-	-	-
57	Germacrene B	1549	1559	[22]	0.23	0.09	1809	1814	[25]	0.16	0.06
58	*trans*-Calamenene	-	-	-	-	-	1821	1821	[29]	0.40	0.13
59	*epi*-Cubebol	-	-	-	-	-	1889	1899	[32]	0.18	0.04
60	α-Calacorene	-	-	-	-	-	1904	1894	[26]	0.13	0.01
61	Palustrol	1563	1567	[22]	0.17	0.04	1918	1915	[23]	0.15	0.02
62	Germacrene D-4-ol	1572	1574	[22]	0.40	0.16	2055	2044	[23]	0.32	0.05
63	1,5-Epoxy-salvial(4)14-ene	-	-	-	-	-	1930	1912	[32]	0.12	0.03
64	Caryophyllene oxide	1575	1582	[22]	0.59	0.09	1971	1967	[23]	0.37	0.07
65	Ledol	1597	1602	[22]	0.45	0.23	2026	2017	[23]	0.60	0.05
66	Cubenol	-	-	-	-	-	2031	2023	[26]	0.33	0.07
67	Silphiperfol-6-en-5-one	1613	1624	[22]	0.29	0.21	2100	2131	[24]	0.39	0.16
68	1-*epi*-Cubenol	1623	1627	[22]	0.16	0.05	2065	2048	[29]	0.23	0.15
69	Spathulenol	-	-	-	-	-	2155	2144	[27]	0.36	0.02
70	*epi*-α-Cadinol	1639	1638	[22]	0.15	0.12	2179	2167	[23]	0.19	0.05
71	*epi*-α-Muurolol	1642	1640	[22]	0.17	0.10	2195	2196	[27]	0.29	0.07
72	α-Muurolol (=Torreyol)	1645	1644	[22]	0.25	0.15	2185	2178	[29]	0.17	0.01
73	*epi*-α-Bisabolol	-	-	-	-	-	2210	2218	[25]	0.16	0.05
74	α-Cadinol	1653	1652	[22]	0.43	0.05	2244	2255	[27]	0.50	0.15
Monoterpene hydrocarbons (%)					34.53					35.61	
Oxygenated monoterpenes (%)					13.21					9.92	
Sesquiterpene hydrocarbons (%)					45.34					44.80	
Oxygenated sesquiterpenes (%)					3.24					4.17	
Other compounds (%)					0.95					1.39	
Total (%)					97.27					96.06	

LRI ^a^, Linear retention index calculated; LRI ^b^, Linear retention index from Reference; Ref, References; % Percentage and SD Standard Deviation, both values were conveyed as means of three determinations.

**Table 2:** Enantioselective analysis of *D. juniperinum* essential oil.

Enantiomeric Compounds	LRI ^a^	Enantiomeric Distribution (%)	*ee* (%) ± SD
(+)-α-Thujene	921	21.83	56.34 ± 0.12
(−)-α-Thujene	924	78.17	
(+)-α-pinene	930	100	100 ± 0.01
(+)-β-Pinene	958	3.39	93.23 ± 1.92
(−)-β-Pinene	966	96.61	
(+)-Sabinene	984	26.08	47.84 ± 2.12
(−)-Sabinene	995	73.92	
(+)-4-Terpineol	1279	71.56	43.11 ± 0.98
(−)-4-Terpineol	1288	28.44	
(+)-Germacrene D	1468	7.69	84.62 ± 0.13
(−)-Germacrene D	1474	92.31	

LRI ^a^, Linear retention index calculated; *ee* (%) ± SD, percentage of excess enantiomeric ± standard deviation values were conveyed as means of three determinations.

**Table 3:** AChE and BuChE inhibition of *D. juniperinum* essential oil.

Sample	AChE	BuChE
	IC_50_ (µg/mL) ± SD	
*D. juniperinum*	67.20 ± 7.10	89.00 ± 9.90
*Donepezil*	0.04 ± 0.01	3.60 ± 0.20

IC_50_, Half maximal inhibition concentration expressed as µg/mL.

**Table 4:** Antioxidant activity of *D. juniperinum* essential oil.

Sample	ABTS	DPPH
	SC_50_ (µg/mL—µM *) *±* SD	
*D. juniperinum*	127.03 ± 0.58	>1000
Trolox *	23.27 ± 1.05	29.99 ± 1.04

SC_50_, Half scavenging capacity expressed as µg/mL—µM *.

## Data Availability

Not applicable.

## References

[1] The Plant List. Compositae. http://www.theplantlist.org/.

[2] Stevens P.F. Angiosperm Phylogeny Website Version 14. http://www.mobot.org/MOBOT/research/APweb/.

[3] Naim D.M., Mahboob S. (2020). Molecular identification of herbal species belonging to genus *Piper* within family Piperaceae from northern Peninsular Malaysia. J. King Saud. Univ. Sci..

[4] Valarezo E., Aguilera-Sarmiento R., Meneses M.A., Morocho V. (2021). Study of Essential Oils from Leaves of Asteraceae Family Species *Ageratina dendroides* and *Gynoxys verrucosa*. J. Essent. Oil Bear. Plants.

[5] Panero J.L., Crozier B.S. (2016). Macroevolutionary dynamics in the early diversification of Asteraceae. Mol. Phylogenet. Evol..

[6] Del Vitto L.A., Petenatti E.M. (2009). Asteraceae of economic and environmental importance: First part. Morphological and taxonomic synopsis, environmental importance and plants of industrial value. Multequina.

[7] Oliveira Amorim V., Bautista Pousada H. (2016). Asteraceae da Ecorregião Raso da Catarina, Bahia, Brasil. Rodriguésia.

[8] Encyclopedia Britannica. Asteraceae: Plant Family. https://www.britannica.com/.

[9] Rivero-Guerra A.O. (2020). Diversity of endemic species of Asteraceae (Compositae) in the flora of Ecuador. Collect. Bot..

[10] Abad M.J., Bedoya L.M., Bermejo P., Rai M.K., Kon K.V. (2013). Essential Oils from the Asteraceae Family Active against Multidrug-Resistant Bacteria. Fighting Multidrug Resistance with Herbal Extracts, Essential Oils and Their Components.

[11] Hama J.R., Strobel B.W. (2021). Occurrence of pyrrolizidine alkaloids in ragwort plants, soils and surface waters at the field scale in grassland. Sci. Total Environ..

[12] Gupta M.P., Arias T.D., Williams N.H., Bos R., Tattje D.H.E. (1985). Safrole, the main component of the essential oil from *Piper auritum* of Panama. J. Nat. Prod..

[13] Kemprai P., Protim Mahanta B., Sut D., Barman R., Banik D., Lal M., Proteem Saikia S., Haldar S. (2020). Review on safrole: Identity shift of the ‘candy shop’ aroma to a carcinogen and deforester. Flavour Frag. J..

[14] Jørgesen P.M., León-Yáñez S. Catalogue of the Vascular Plants of Ecuador. http://legacy.tropicos.org/ProjectAdvSearch.aspx?projectid=2.

[15] León-Yánez S., Valencia R., Pitmam N., Endara L., Ulloa Ulloa C., Navarrete H. Libro Rojo de Plantas Endémicas del Ecuador. https://bioweb.bio/floraweb/librorojo/home.

[16] The Plant List. Diplostephium. http://www.theplantlist.org/.

[17] Vargas O., Madriñán S. (2006). Clave para la identificación de las especies del género *Diplostephium* (Asteraceae, Astereae) en Colombia. Rev. Real Acad. Cienc. Exactas Fis. Nat..

[18] Vargas O.M., Madriñán S. (2012). Preliminary Phylogeny of *Diplostephium* (Asteraceae): Speciation Rate and Character Evolution. Lundellia.

[19] Andrade J.M., Lucero-Mosquera H., Armijos C. (2017). Ethnobotany of indigenous Saraguros: Medicinal plants used by community healers “Hampiyachakkuna” in the San Lucas Parish, Southern Ecuador. Biomed. Res. Int..

[20] Vargas Oscar M. (2011). A Nomenclator of *Diplostephium* (Asteraceae: Astereae): A List of Species with Their Synonyms and Distribution by Country. Lundellia.

[21] Mestanza-Ramón C., Henkanaththegedara S.M., Vásconez Duchicela P., Vargas Tierras Y., Sánchez Capa M., Constante Mejía D., Jimenez Gutierrez M., Charco Guamán M., Mestanza Ramón P. (2020). In-Situ and Ex-Situ Biodiversity Conservation in Ecuador: A Review of Policies, Actions and Challenges. Diversity.

[22] Adams R.P. (2007). Identification of Essential Oil Components by Gas, Chromatography/Mass Spectrometry.

[23] Salinas M., Bec N., Calva J., Ramírez J., Andrade J.M., Larroque C., Vidari G., Armijos C. (2020). Chemical composition and anticholinesterase activity of the essential oil from the Ecuadorian plant *Salvia pichinchensis* Benth. Rec. Nat. Prod..

[24] Demirci B., Başer K., Aytaç Z., Khan S.I., Jacob M.R., Tabanca N. (2018). Comparative study of three *Achillea* essential oils from eastern part of Turkey and their biological activities. Rec. Nat. Prod..

[25] Armijos C., Matailo A., Bec N., Salinas M., Aguilar G., Solano N., Calva J., Ludeña C., Larroque C., Vidari G. (2020). Chemical composition and selective BuChE inhibitory activity of the essential oils from aromatic plants used to prepare the traditional Ecuadorian beverage *horchata lojana*. J. Ethnopharmacol..

[26] Paolini J., Muselli A., Bernardini A.F., Bighelli A., Casanova J., Costa J. (2007). Thymol derivatives from essential oil of *Doronicum corsicum* L.. Flavour Fragr. J..

[27] Viljoen A.M., Kamatou G.P., Coovadia Z.H., Özek T., Başer K.H.C. (2010). Rare sesquiterpenes from South African *Pteronia* species. S. Afr. J. Bot..

[28] Capetanos C., Saroglou V., Marin P.D., Simić A., Skaltsa H.D. (2007). Essential oil analysis of two endemic *Eryngium* species from Serbia. J. Serb. Chem. Soc..

[29] Montalván M., Peñafiel M.A., Ramírez J., Cumbicus N., Bec N., Larroque C., Bicchi C., Gilardoni G. (2019). Chemical composition, enantiomeric distribution, and sensory evaluation of the essential oils distilled from the Ecuadorian species *Myrcianthes myrsinoides* (Kunth) Grifo and *Myrcia mollis* (Kunth) dc. (Myrtaceae). Plants.

[30] Başer K.H.C., Demirci B., Kirimer N.E., Satil F., Tümen G. (2002). The essential oils of *Thymus migricus* and *T. fedtschenkoi* var. handelii from Turkey. Flavour Fragr. J..

[31] Maggio A., Bruno M., Guarino R., Senatore F., Ilardi V. (2016). Contribution to a Taxonomic Revision of the *Sicilian Helichrysum* Taxa by PCA Analysis of Their Essential-Oil Compositions. Chem. Biodivers..

[32] Özek G., Bedir E., Tabanca N., Ali A., Khan I.A., Duran A., Baser K., Özek T. (2018). Isolation of eudesmane type sesquiterpene ketone from *Prangos heyniae* H. Duman & MF Watson essential oil and mosquitocidal activity of the essential oils. Open Chem..

[33] Carrillo-Hormaza L., Mora C., Alvarez R., Alzate F., Osorio E. (2015). Chemical composition and antibacterial activity against *Enterobacter cloacae* of essential oils from Asteraceae species growing in the Páramos of Colombia. Ind. Crops. Prod..

[34] Him A., Ozbek H., Turel I., Oner A.C. (2008). Antinociceptive activity of alpha-pinene and fenchone. Pharmacologyonline.

[35] Kim D.S., Lee H.J., Jeon Y.D., Han Y.H., Kee J.Y., Kim H.J., Shin H.J., Kang J., Lee B.S., Kim S.J. (2015). Alpha-Pinene Exhibits Anti-Inflammatory Activity Through the Suppression of MAPKs and the NF-κB Pathway in Mouse Peritoneal Macrophages. Am. J. Chin. Med..

[36] Martin S., Padilla E., Ocete M.A., Galvez J., Jiménez J., Zarzuelo A. (1993). Anti-inflammatory activity of the essential oil of *Bupleurum fruticescens*. Planta Med..

[37] Zhou J.Y., Tang F.D., Mao G.G., Bian R.L. (2004). Effect of alpha-pinene on nuclear translocation of NF-kappa B in THP-1 cells. Acta Pharmacol. Sin..

[38] Ahmad A., Husain A., Mujeeb M., Khan S.A., Najmi A.K., Siddique N.A., Damanhouri Z.A., Anwar F. (2013). A review on therapeutic potential of *Nigella sativa*: A miracle herb. Asian Pac. J. Trop. Biomed..

[39] Singh H.P., Batish D.R., Kaur S., Arora K., Kohli R.K. (2006). alpha-Pinene inhibits growth and induces oxidative stress in roots. Ann. Bot..

[40] Zore G.B., Thakre A.D., Rathod V., Karuppayil S.M. (2011). Evaluation of anti-Candida potential of geranium oil constituents against clinical isolates of *Candida albicans* differentially sensitive to fluconazole: Inhibition of growth, dimorphism and sensitization. Mycoses.

[41] Quintans-Júnior L., Moreira J.C.F., Pasquali M.A.B., Rabie S.M.S., Pires A.S., Schröder R., Rabelo T.K., Santos J.P.A., Lima P.S.S., Cavalcanti S.C.H. (2013). Antinociceptive activity and redox profile of the monoterpenes (+)-camphene, p-cymene, and geranyl acetate in experimental models. ISRN Toxicol..

[42] Mozuraitis R., Stranden M., Ramirez M.I., Borg-Karlson A.K., Mustaparta H. (2002). (-)-Germacrene D increases attraction and oviposition by the tobacco budworm moth *Heliothis virescens*. Chem. Senses.

[43] Lis-Balcnin M., Ochocka R.J., Deans S.G., Asztemborska M., Hart S. (1999). Differences in bioactivity between the enantiomers of α-pinene. J. Essent. Oil Res..

[44] Brenna E., Fuganti C., Serra S. (2003). Enantioselective perception of chiral odorants. Tetrahedron Asymmetry.

[45] Thomsen T., Kewitz H. (1990). Selective inhibition of human acetylcholinesterase by galanthamine in vitro and in vivo. Life Sci..

[46] Blanco-Silvente L., Castells X., Saez M., Barceló M.A., Garre-Olmo J., Vilalta-Franch J., Capellà D. (2017). Discontinuation, Efficacy, and Safety of Cholinesterase Inhibitors for Alzheimer’s Disease: A Meta-Analysis and Meta-Regression of 43 Randomized Clinical Trials Enrolling 16 106 Patients. Int. J. Neuropsychopharmacol..

[47] Orhan I., Kartal M., Şener B. (2008). Activity of Essential oils and individual components against Acetyland butyrylcholinesterase. Z. Naturforsch.

[48] Leporini M., Bonesi M., Loizzo M.R., Passalacqua N.G., Tundis R. (2020). The essential oil of *Salvia rosmarinus* Spenn. from Italy as a Source of Health-Promoting Compounds: Chemical Profile and Antioxidant and Cholinesterase Inhibitory Activity. Plants.

[49] Aazza S., Lyoussi B., Miguel M.G. (2011). Antioxidant and antiacetylcholinesterase activities of some commercial essential oils and their major compounds. Molecules.

[50] Miyazawa M., Yamafuji C. (2005). Inhibition of acetylcholinesterase activity by bicyclic monoterpenoids. J. Agric. Food Chem..

[51] Rodriguez-Aguirre O.E., Torrenegra-Guerrero R.D.T. (2018). Flavonoids, Terpenes and the Anti-Oxidant Activity of *Diplostephium phylicoides* (HBK) Wedd. Indian J. Sci. Technol..

[52] Wang C.Y., Chen Y.W., Hou C.Y. (2019). Antioxidant and antibacterial activity of seven predominant terpenoids. Int. J. Food Prop..

[53] Pham-Huy L.A., He H., Pham-Huy C. (2008). Free radicals, antioxidants in disease and health. Int. J. Biomed. Sci..

[54] Rolnik A., Olas B. (2021). The Plants of the Asteraceae Family as Agents in the Protection of Human Health. Int. J. Mol. Sci..

[55] Nikolić M., Stevović S. (2015). Family Asteraceae as a sustainable planning tool in phytoremediation and its relevance in urban areas. Urban For. Urban Green..

[56] Oliveira M., Brugnera D., Cardoso M., Guimarães L., Piccoli R. (2011). Rendimento, composição química e atividade antilisterial deóleos essenciais de espécies de *Cymbopogon*. Rev. Bras. Plantas Med..

[57] Calva J., Bec N., Gilardoni G., Larroque C., Cartuche L., Bicchi C., Montesinos J.V. (2017). Acorenone B: AChE and BChE Inhibitor as a Major Compound of the Essential Oil Distilled from the Ecuadorian Species *Niphogeton dissecta* (Benth.) J.F. Macbr. Pharmaceuticals.

[58] Van Den Dool H., Kratz P.D. (1963). A generalization of the retention index system including linear temperature programmed gas—Liquid partition chromatography. J. Chromatogr..

[59] Villalta G., Salinas M., Calva J., Bec N., Larroque C., Vidari G., Armijos C. (2021). Selective BuChE Inhibitory Activity, Chemical Composition, and Enantiomeric Content of the Essential Oil from *Salvia leucantha* Cav. Collected in Ecuador. Plants.

[60] Gilardoni G., Matute Y., Ramírez J. (2020). Chemical and Enantioselective Analysis of the Leaf Essential Oil from *Piper coruscans* Kunth (Piperaceae), a Costal and Amazonian Native Species of Ecuador. Plants.

[61] Calva J., Cartuche L., González S., Montesinos J.V., Morocho V. (2022). Chemical composition, enantiomeric analysis and anticholinesterase activity of *Lepechinia betonicifolia* essential oil from Ecuador. Pharm. Biol..

[62] Ellman G.L., Courtney K.D., Andres V., Featherstone R.M. (1961). A new and rapid colorimetric determination of acetylcholinesterase activity. Biochem. Pharmacol..

[63] Thaipong K., Boonprakob U., Crosby K., Cisneros-Zevallos L., Hawkins Byrne D. (2006). Comparison of ABTS, DPPH, FRAP, and ORAC assays for estimating antioxidant activity from guava fruit extracts. J. Food Compos. Anal..

[64] Arnao M.B., Cano A., Acosta M. (2001). The hydrophilic and lipophilic contribution to total antioxidant activity. Food Chem..

